# Deep vein thrombosis in donor or recipient veins encountered during lower extremity reconstruction with a free anterolateral thigh perforator flap: How do we deal with it?

**DOI:** 10.3389/fsurg.2022.985245

**Published:** 2022-09-28

**Authors:** Seong-Ho Jeong, Sik Namgoong, Eun-Sang Dhong, Seung-Kyu Han

**Affiliations:** Department of Plastic Surgery, Korea University Guro Hospital, Seoul, South Korea

**Keywords:** deep vein thrombosis, lower extremity reconstruction, anterolateral thigh perforator flap, computed tomographic venography, algorithmic approach, venous thromboembolism (VTE)

## Abstract

**Background:**

The free anterolateral thigh perforator (ALTP) flap has been successfully adopted to reconstruct traumatic soft tissue defects in the lower extremities. However, the occurrence of deep vein thrombosis (DVT) in donor or recipient veins has been overlooked, and there has been no reliable guideline to manage it. Therefore, in this study, we review our cases where the ALTP flaps were transferred to traumatic lower limbs even though DVT was found in the pedicle or recipient veins. Furthermore, based on our experiences, we suggest an algorithmic approach for dealing with DVT.

**Patients and methods:**

This study included 108 patients who underwent lower extremity reconstruction using a free ALTP flap between January 2014 and January 2021. All medical records were reviewed, including preoperative assessment data, intraoperative findings, and postoperative complications. Notably, when DVT was found in both the donor and recipient veins, we thoroughly assessed operative findings, surgical solutions, and final outcomes.

**Results:**

Sixty-one of 108 (56.4%) patients underwent computed tomographic venography (CTV) preoperatively, revealing DVT in 11 of these 61 (18%) patients. Three of these 11 patients had iliofemoral DVT, and surgery was delayed more than two weeks after detection. The remaining eight patients had calf DVT and underwent free ALTP flap transfer as scheduled. Conversely, 47 of 108 (43.6%) patients did not undergo CTV, and an occult DVT was found in five of these 47 (10.6%) patients. In two of these five patients, free flap surgery was replaced with amputation and local flap coverage. In the remaining three patients and one patient with an occult DVT that was not found on CTV, the free ALTP flap transfer was carried out. In 15 patients with DVT, free ALTP flap transfer was performed using various alternative methods for venorrhaphy. Consequently, all flaps survived, with partial necrosis occurring in two patients.

**Conclusion:**

If DVT-affected veins are appropriately managed, the free ALTP flap can be successfully transferred to the traumatic lower limb even when DVT occurs in donor or recipient veins. The author's algorithm can help surgeons overcome the insufficiency of veins for pedicle anastomosis due to DVT and avoid postoperative thromboembolic complications.

## Introduction

Deep vein thrombosis (DVT) is the formation of blood clots within the deep venous system of the body, most frequently in the lower extremities. It is well known that DVT can lead to fatal systemic complications such as pulmonary embolism and heart attack (1). However, limitations on using veins due to obstruction by DVT in microsurgical reconstruction have not been highlighted. Concerning free flap transfer, DVT can be an obstacle to achieving successful outcomes because of a scarcity of healthy recipient veins (2).

DVT tends to occur more commonly in patients with lower extremity trauma (3). This is because multiple risk factors for DVT, such as blood transfusion, direct venous trauma, hypercoagulable states, and immobilization, could exist simultaneously (4). When soft tissue defects are accompanied by severe lower extremity trauma, such as Gustilo type IIIB or IIIC open fractures, the presence of DVT might play a key factor in the success or failure of the management. Since these injuries usually require free tissue transfer, which takes a long time and involves immobilization, the risk of a perioperative fatal thromboembolic event or a free flap failure due to venous obstruction increases.

The anterolateral thigh perforator (ALTP) flap has been popularly used to reconstruct traumatic soft tissue defects in the lower extremities and has shown overall good results (5, 6). Nevertheless, when the free ALTP flap is employed to reconstruct massive lower extremity trauma, the incidence of DVT-related complications may be higher than the use of other flaps harvested from locations other than the lower extremities. It is because the pedicle of the ALTP flap generally consists of one artery and two concomitant deep veins, and these deep veins should be anastomosed to other deep veins of recipient vessels in the injured area of the lower extremities. Thus, preoperative screening tests, such as doppler ultrasound (US) and computed tomographic venography (CTV), are recommended for patients with high-risk factors for DVT before free ALTP flap transfer to the lower extremities (7).

Although these tests can help detect DVT preoperatively, they cannot perfectly reveal its presence. Besides, in some cases, reconstructive procedures must be attempted despite recognizing the existence of DVT because there is no more time to delay due to time lost from previous inadequate management. Therefore, microsurgeons have a chance of encountering DVT, intentionally or unintentionally, during lower extremity reconstruction with free ALTP flaps (4, 8, 9).

Several researchers have published their experiences dealing with DVT concerning free flap procedures (2, 4, 8, 10–14). Most of them focused on the systemic adverse effects of DVT on venous anastomosis in free flap transfer. Nevertheless, there are few reports on the feasibility of using the veins occupied by DVT as recipient veins locally. Furthermore, the reports regarding DVT in donor veins are even fewer, and all of them are case reports on occult DVT in peroneal veins of free osteocutaneous fibula flaps (10–13). So far, no clinical guidelines are available to cope with DVT in veins to be used for pedicle anastomosis during free ALTP flap transfer.

We have successfully transferred free ALTP flaps for traumatic lower extremity reconstruction in patients with DVT in either donor or recipient veins. So, herein, we describe our experience treating DVT in this rare but critical situation. The primary goal of this study was to determine the incidence of DVT in donor or recipient veins in lower extremity reconstruction cases using free ALTP flaps. The secondary objective was to suggest a basic surgical algorithm for dealing with DVT encountered in free ALTP flap reconstruction.

## Patients and methods

Between January 2014 and September 2021, free ALTP flap transfer was performed for traumatic lower extremity reconstruction in 121 cases in 118 patients by the first author (S.H.J) at a single institution. The surgical procedures were consistent with the ethical guidelines of the 1975 Declaration of Helsinki. Thirteen cases were excluded because of inadequate medical records or loss of follow-up, leaving 108 cases (91 men and 17 women). The patients' ages ranged from 18 to 74 years, with an average of 45.6 years. All medical records of these 108 patients were retrospectively reviewed. Patients' demographics, preoperative assessment data, intraoperative findings, postoperative complications, and overall surgical outcomes were recorded. In particular, an in-depth analysis of cases in which DVT was found in the donor or recipient veins was carried out. In these cases, the operative findings of DVT, the surgical solutions, and the final outcomes were all thoroughly reviewed (Tables 1, 2).

**Table 1:** Clinical features of the patients with DVT identified by preoperative CTV for a free ALTP flap transfer.

Patient No	Sex/Age	Interval to surgery	Defect location	Affected vein by DVT on CTV	DVT location^a^	DVT feature^a^	Surgical solutions	Free Flap outcome
1	M/33	13 days	Medial	IFV involvement	N/A	N/A	Delayed free ALTP flap transfer (3weeks later) following chemoprophylaxis and NPWT	Total survival
2	M/43	10 days	Lateral	IFV involvement	N/A	N/A	Delayed free ALTP flap transfer (2weeks later) following chemoprophylaxis and NPWT	Total survival
3	M/56	21 days	Medial	IFV involvement	N/A	N/A	Delayed free ALTP flap transfer (3weeks later) following IVC filter placement and NPWT	Total survival
4	M/36	8 days	Medial	PTV	R, PTV(1)	Cont	Nonuse of affected PTV(1) Use of GSVwith vein graft and unaffected PTV(1)	Total survival
5	F/56	12 days	Lateral	ATV	R, ATV(1)	Seg	Resection of ATV(1) segment including thrombus Use of affected ATV(1) and unaffected ATV(1)	Partial necrosis
6	M/29	9 days	Medial	PTV	R, PTV(1)	Cont	Nonuse of affected PTV(1) Use of only single unaffected PTV(1)	Total Survival
7	M/55	13 days	Medial	PTV	R, PTV(2)	Cont	Delayed free ALTP flap transfer using ATV (2weeks later) following chemoprophylaxis and NPWT	Total Survival
8	M/38	14 days	Lateral	ATV	R, ATV(1)	Seg	Resection of ATV(1) segment including thrombus Use of affected ATV(1) and unaffected ATV (1)	Total Survival
9	F/46	11 days	Lateral	PTV	N/A	N/A	Considering defect's location, Use of ATV(2) initially instead of PTV seemed to be affected by DVT	Total Survival
10	M/56	17 days	Medial	ATV	N/A	N/A	Considering defect's location, Use of PTV (1) and GSV initially instead of ATV seemed to be affected by DVT	Total Survival
11	M/51	14 days	Medial	ATV, PTV	N/A	N/A	Delayed free ALTP flap transfer (3weeks later) with chemoprophylaxis / NPWT	Total Survival

DVT, deep vein thrombosis; CTV, CT venography; ALTP, anterolateral thigh perforator; No, number; IFV, iliofemoral vein; NPWT, negative pressure wound therapy; R, recipient; PTV, posterior tibial vein (Concomitant veins of posterior tibial artery); Cont, continuous; GSV, great saphenous vein; ATV, anterior tibial vein (Concomitant veins of anterior tibial artery); Seg, segmental.

^a^ Intraoperative findings regarding DVT (The numbers in parenthesis refers to the number of veins affected by DVT).

**Table 2:** Clinical features of the patients with DVT encountered intraoperatively during free ALTP flap transfer.

Patient No	Sex/Age	Interval to surgery	Defect location	Affected vein on CTV	DVT location^a^	DVT feature^a^	Surgical solutions	Free Flap outcome
12	M/33	14 days	Lateral	None	R, ATV(1)	Cont	Nonuse of the affected vein and Single venous anastomosis with unaffected ATV(1) Complementary local flap coverage	Total Survival
13	M/74	6 days	Medial	N/A	R, PTV(2)	Cont	Nonuse of the affected vein Amputation	N/A
14	M/32	5 days	Medial	N/A	R, ATV(2)	Cont	Nonuse of the affected vein Switch to local flap coverage	N/A
15	M/25	4 days	Lateral	N/A	D, LCFAd-CV(2)	6 cm	Removal of the total LCFAd segment of the pedicle Use of only perforators as flap pedicles	Partial necrosis
16	M/70	5 days	Lateral	N/A	D, LCFAd-CV(1)	2 cm	Simple removal of only the thrombus from the vein Use of the whole LCFAd vein as a donor vein	Total survival
17	M/52	6 days	Lateral	N/A	D, LCFAt-CV(1)	3 cm	Removal of the vein segment including thrombus Use of the remaining LCFAt vein as a donor vein	Total survival

DVT, deep vein thrombosis; CTV, computed tomographic venography; ALTP, anterolateral thigh perforator; No, number; R, recipient; ATV, anterior tibial vein (Concomitant veins of anterior tibial artery); Cont, continuous; PTV, posterior tibial vein (Concomitant veins of posterior tibial artery); D, Donor; LCFAd-CV, Concomitant vein in the descending branch of lateral circumflex femoral artery; LCFAt-CV, Concomitant vein in the transverse branch of the lateral circumflex femoral artery.

^a^ Intraoperative findings regarding DVT (The numbers in parenthesis refers to the number of veins affected by DVT).

### Surgical techniques to deal with DVT

With a hand-held Doppler, preoperative mapping of perforators in the anterolateral thigh area was performed after drawing the guideline from the anterior superior iliac spine to the upper lateral patella the day before surgery. The surgery was carried out under general anesthesia with the patients in a supine position. At first, in the injured lower leg area, the orthopedic surgeon debrided all contaminated and necrotic tissues, including bone and soft tissue, and tried to fixate fractures temporarily with or without antibiotic-loaded bone cement. Finally, soft-tissue defects were created following debridement. Next, to explore and prepare the recipient vessels for free tissue transfer, the plastic surgeon made an oblique incision from the medial or lateral margin of the defect to the posterior side of the leg to expose the vessels. Then, with retraction of the calf muscles, the anterior tibial artery (ATA) or posterior tibial artery (PTA) and its concomitant veins, which are respectively called anterior tibial vein (ATV) or posterior tibial vein (PTV), were exposed and isolated from the muscles. During the preparation of the recipient vessels, DVT was found in these concomitant veins in some cases.

Although concomitant veins can merge into one vein or divide into multiple veins, they usually consist of two veins. When the DVT was found in both two concomitant veins, free tissue transfer was not performed because of the high risk of venous thromboembolism in the flap after surgery. Instead, if available, local flap coverage as a substitute was attempted, or delayed surgical management with negative pressure wound therapy (NPWT) was considered. However, amputation was considered in aged patients who seemed unable to undergo additional surgeries due to unstable general conditions caused by trauma and underlying diseases. When DVT was found in only one concomitant vein, the continuity was investigated by tracing the vein proximally. In cases where the DVT was present continuously to the most proximal point of the operative field, the veins were regarded as unusable due to loss of drainage function. Thus, single venous anastomosis using one intact concomitant vein or double venous anastomosis, including one intact concomitant vein and one superficial vein, if available, was performed in these cases. In contrast, in cases where the DVT was present segmentally in the middle of the vein, the whole vein segment containing the thrombus was removed, and the proximal DVT-free vein was used as a recipient vein. Therefore, double venous anastomosis was attempted in these cases.

After finishing the recipient vessel preparation, a transparent template reflecting the exact shape of the soft-tissue defect was made. Based on this template, the ALTP flap was designed on the thigh with the flap center matching the perforators' location marked preoperatively. An incision was made on the medial border of the flap through the skin, subcutaneous fat, and fascia over the vastus lateralis muscle. Next, the flap was elevated from the medial to the lateral direction in the subfascial plane to explore the perforators. Once reliable perforators were found, they were isolated from the adjacent soft tissues and dissected to the origin of the lateral circumflex femoral artery (LCFA) to achieve sufficient pedicle length. Before severing the pedicle, the artery and concomitant veins were separated from each other to facilitate anastomosis. During this procedure, DVT was found incidentally in some cases. Once the DVT was identified, the patency of the veins was confirmed by microscopic examination. When the DVT was less than 3 cm in length, the whole vein was used for anastomosis after simple removal of the thrombus. Thrombus removal was tried only in the pedicle veins of ALTP flaps because the veins are freely movable to enable adequate inspection of the lumen and manipulation of the thrombus. In contrast, it was not attempted in recipient veins due to the limited mobility of the veins and the ambiguous boundary of the thrombus. However, when the length of DVT exceeded 3 cm, the whole vein segment containing the thrombus was resected, and the remaining DVT-free vein was used as a donor vein because complete removal of DVT longer than 3 cm in length was very tough, according to our institutional experience.

## Results

Of the 108 cases where the soft-tissue reconstruction using a free ALTP flap was performed, 82 cases (75.9%) had open tibia fractures accompanied by soft tissue damage at the time of injury. In comparison, 20 cases (18.5%) had initially closed fractures with subsequent bone exposure following wound breakdown. Six cases (5.5%) had only extensive soft tissue injuries without fractures. The mean time interval between the initial injury and free flap surgery was 10.1 days (range, 4–32 days). A preoperative screening test to detect DVT using CTV was performed when the delay from injury to free flap exceeded seven days. Sixty-one of 108 (56.4%) patients underwent CTV, revealing DVT in 11 of 61 (18%) patients (Table 1). In three of these 11 patients, iliofemoral DVT was detected, and surgery was delayed for at least two weeks. On the contrary, the remaining eight patients had calf DVT. One of these eight patients suffered from DVT occupying both ATV and PTV, and should have undergone delayed free ALTP flap transfer following adequate chemoprophylaxis for DVT and NPWT. The other seven patients presented DVT in only one vein, either ATV or PTV. For these patients, immediate lower leg reconstruction using free ALTP flap transfer was attempted. Considering the proximity of the vein affected by DVT to the defect, the free ALTP flap transfer was performed, employing the veins unaffected by DVT as recipient veins in two patients and the veins affected by DVT in five patients.

Intraoperative investigation of DVT in these five patients demonstrated that DVT occupied both concomitant veins in one patient and only one concomitant vein in four patients. The patient with DVT in both concomitant veins undertook a delayed free ALTP flap transfer using veins unaffected by DVT two weeks after the first surgical intervention because continuous thrombus occupied both veins. Of the four patients with DVT in only one concomitant vein, two patients had segmental thrombus, and another two had continuous thrombus. In two patients with segmental thrombus, the vein was used as the recipient vein after simply resecting the segment containing the thrombus. For two patients with continuous thrombus, the vein was substituted with the great saphenous vein (GSV) in one patient and not used for venous anastomosis in the other patient. In the latter patient, single venous anastomosis using an unaffected concomitant vein was performed. Of the 11 patients diagnosed with DVT preoperatively, three patients with iliofemoral DVT were initially managed by administering therapeutic doses of low molecular weight heparin (LMWH). The prophylactic dose of LMWH was started in the other eight patients on the day of diagnosis.

Even though DVT was not detected preoperatively by CTV, continuous DVT formed in one concomitant vein was found intraoperatively in one patient, and a single venous anastomosis using an unaffected vein was performed (Table 2. Patient No. 12). Forty-seven of 108 (43.6%) patients did not undergo preoperative CTV, and occult DVT was found in the operative field in five out of 47 (10.6%) patients (Table 2. Patient No. 13–17). In these patients, DVT was discovered in the recipient veins of the ALTP flap in two patients and in the donor veins in three patients. In two patients with DVT in the recipient veins, both concomitant veins were obstructed by continuous thrombus. As a result of the patient's poor general condition and inability to withstand additional surgical stress, amputation was performed on one patient. Besides, local flap coverage was attempted for the other patient, and successful reconstruction was achieved. In three patients with DVT in the donor veins, the veins occupied by DVT were re-used after removing the thrombus in one patient and after resecting the venous segment containing the thrombus in two patients (Table 2).

Free ALTP flap transfer was attempted in all 108 patients in this study. DVT was identified in 17 (15.7%) of these patients preoperatively or intraoperatively. However, as mentioned above, the surgical procedure was not completed in two patients with both concomitant veins in recipient vessels occupied by DVT. Therefore, the free ALTP flap was transferred to 106 patients and, in particular, to 15 patients with DVT. Total loss of the ALTP flap occurred in one out of 106 patients. However, all ALTP flaps survived in all 15 patients with DVT. Partial necrosis was observed in six of the 106 patients (5.7%) and in two of the 15 patients with DVT. This resulted in revisional surgery in five patients.

## Case reports

### Case 1 (patient No. 4)

**Figure 1:**
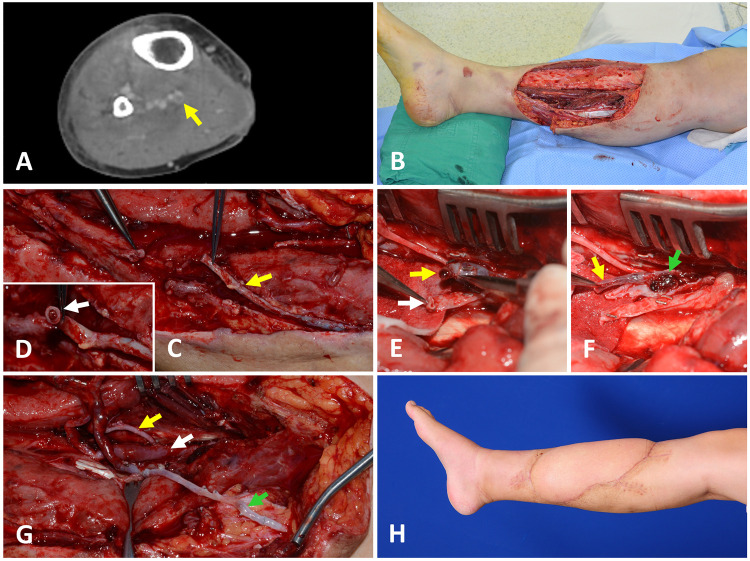
A case presenting the DVT in one posterior tibial vein encountered during the preparation of the recipient vessels for the free ALTP flap transfer (**A**) preoperative CTV examination showed an intraluminal filling defect at the posterior tibial vein, which implies the presence of DVT. (**B**) A large soft tissue defect was created after radical debridement. (**C**) During the surgical approach to the recipient vessels, the GSV was preserved and isolated from fat tissue to use as an alternative venous drainage route if required (yellow arrow). (**D**) However, the lumen of GSV was obstructed by a thrombus (white arrow). So, a DVT-free proximal GSV was prepared after resecting the vein segment containing the thrombus. (**E**) Surgical exposure of the recipient vessels revealed that one posterior tibial vein was obstructed by DVT (yellow arrow), and the other was intact (white arrow). (**F**) DVT in one posterior tibial vein (yellow arrow) was present continuously and occupied the whole vein exposed in the operative field (green arrow). This vein was unavailable for use as a recipient vein. (**G**) Finally, one pedicle vein of the ALTP flap was anastomosed to one posterior tibial vein that was not affected by DVT (white arrow), and the other flap vein was connected to the prepared GSV (green arrow) after the pedicle artery was anastomosed to the posterior tibial artery in an end-to-side fashion (yellow arrow). (**H**) The transferred flap completely survived. DVT, deep vein thrombosis; CTV, computed tomographic venography; ALTP, anterolateral thigh perforator; GSV, great saphenous vein.

A 36-year-old male patient suffered a crush injury without fractures in the right anterior tibial area caused by a motorcycle accident. After initial surgical debridement, wide soft tissue defects were created (Figure 1A). On the day before free flap surgery, at seven days post-injury, a screening CTV was performed, demonstrating DVT in the posterior tibial vein. However, posterior tibial vessels were selected as the recipient vessels because they are readily accessible through an open wound, and the DVT seemed to be easily removed due to its short length in the CTV. The GSV was preserved and dissected during the surgical exposure of the recipient vessels (Figure 1B). However, the lumen of the GSV was obstructed by a thrombus (Figure 1C). Therefore, the vein segment containing the thrombus was resected, and the DVT-free proximal GSV was prepared for anastomosis. DVT was also identified in only one PTV (Figure 1D). Unlike preoperative assumption, DVT was present continuously and occupied the whole vein exposed in the operative field (Figure 1E). Thus, we decided to use GSV as an alternative recipient vein. After harvesting an ALTP flap (21 × 10 cm^2^) from the contralateral thigh, we transferred it to the defect in the lower leg with its artery anastomosed to the PTA in an end-to-side fashion. Additionally, we anastomosed its pedicle veins to the one PTV that was not affected by DVT in an end-to-end fashion and to the GSV with a vein graft (Figure 1F). The flap survived completely (Figure 1G). LMWH was administered to the patient preoperatively and maintained for three months after surgery.

### Case 2 (patient No. 14)

**Figure 2:**
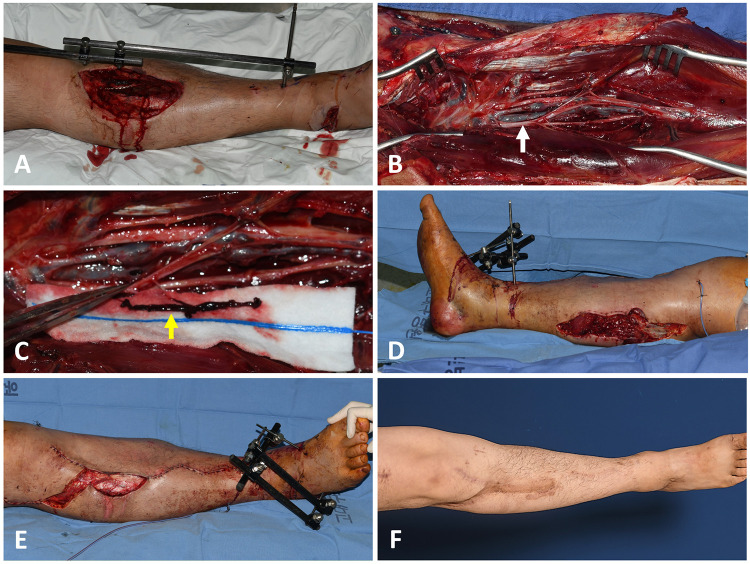
A case showing the DVT found in two anterior tibial veins. (**A**) A 32-year-old male had a soft tissue defect on his right lower leg following an open tibia fracture. (**B**) DVT was found in both the anterior tibial veins. Whole veins exposed in the operative field were filled with DVT (white arrow). (**C**) DVT removal was attempted (yellow arrow), but it was impossible. (**D**) Thus, the planned free transfer of the anterolateral thigh perforator flap was replaced by local flap coverage. At first, a relaxing incision was made on the posterior calf. (**E**) Local soft tissues around the defect were advanced to the defect after undermining. (**F**) The defect was successfully closed with a local flap. DVT, deep vein thrombosis.

A 32-year-old male patient presented with an open wound in the upper anterior tibial area accompanied by an open tibia fracture. Wide soft tissue defects were made after initial debridement and bone fixation (Figure 2A). Early reconstruction was planned because bone and soft tissue injuries did not seem serious, and the patient's general condition was stable. Five days after the injury, a free ALTP flap transfer was attempted using anterior tibial vessels as the recipient vessels. A preoperative DVT screening test was not carried out due to the short time interval between injury and reconstruction. However, unfortunately, the full lengths of both concomitant veins of the ATA were filled with DVT (Figures B,C). Therefore, the planned free ALTP transfer was replaced by local flap coverage. After making a relaxing incision on the posterior side of the calf (Figure 2D), the soft tissues around the defect were undermined and advanced to the defect bilaterally (Figure 2E). Successful reconstruction was achieved (Figure 2F). LMWH was administered to the patient after surgery and maintained for three months.

### Case 3 (patient No. 15)

**Figure 3:**
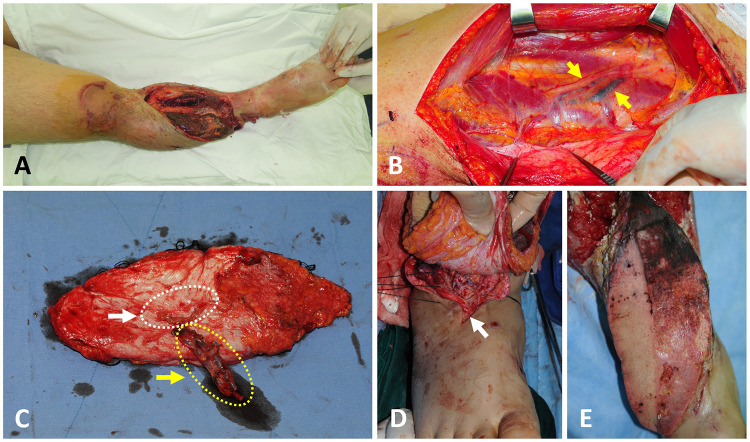
A case showing DVT in two concomitant veins of the DB-LCFA found during the harvest of the anterolateral thigh perforator flap. (**A**) A 25-year-old male sustained severe tibia open fractures accompanied by a soft tissue defect on the right lower leg. (**B**) Surgical exposure of the DB-LCFA reveals dark-colored veins (yellow arrow). (**C**) DVT was identified in the middle of both concomitant veins of DB-LCFA under close inspection of the pedicle. The whole segment of DB-LCFA and its concomitant veins (yellow arrow and dotted line) were resected from the pedicle because DVT could not be removed. Then, only the perforator portion of the pedicle (white arrow and dotted line) was used for anastomosis. (**D**) A vascular anastomosis was performed between the perforator vessels of the flap and dorsalis pedis artery and one of its vena comitantes. Additionally, one perforator vein of the flap was anastomosed to a superficial vein around the recipient site. (**D**) The flap survived with partial necrosis of the proximal portion. DVT, deep vein thrombosis; DB-LCFA descending branch of the lateral circumflex femoral artery.

A 25-year-old man had an open comminuted tibia fracture in his right lower leg following a motorcycle accident (Figure 3A). At first debridement surgery, after thorough removal of necrotic tissues, including skin, muscle, and bone segments, an extensive soft-tissue defect was created. So, early reconstruction of soft tissues was tried to prevent devastating infection and the progression of bone necrosis. On the fourth day after trauma, an ALTP flap (22 × 9 cm^2^) was harvested from the contralateral thigh. Even though the concomitant veins of the descending branch of LCFA appeared much darker than usual (Figure 3B), the thrombus could not be identified at the severing point of the pedicle. However, a DVT, about 6 cm long, was discovered in the middle of both veins during close inspection of the harvested flap (Figure 3C). It was impossible to remove the thrombus from the venous lumen, so the whole segment of the descending branch of the LCFA was removed from the pedicle, and only the perforator part was used as the pedicle of the flap. Arterial anastomosis was performed between the perforator artery and the dorsalis pedis artery with reverse arterial flow, and venous anastomosis was performed between the perforator veins and two recipient veins, including a concomitant vein and a superficial vein (Figure 3D). The flap survived with partial necrosis, which required an additional wound care procedure. Anticoagulation treatment with LMWH was commenced after surgery for three months.

### Case 4 (patient No. 16)

**Figure 4:**
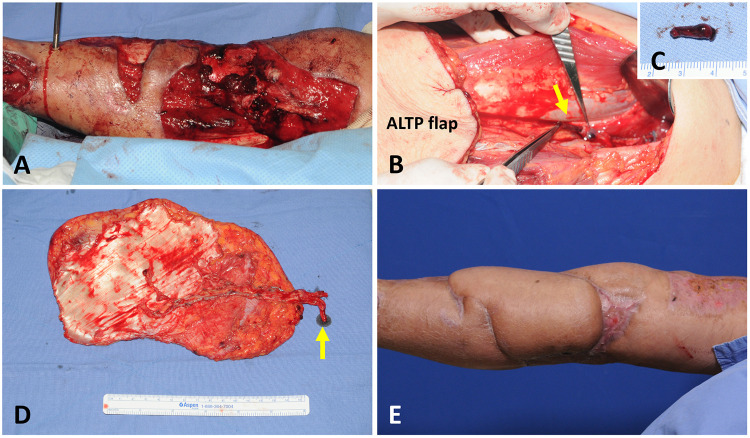
A case demonstrating DVT in one concomitant vein of DB-LCFA was discovered during the elevation of the ALTP flap. (**A**) A 70-year-old male suffered from a massive wound in the upper half of the calf that extended to the knee area. (**B**) DVT was found at the severed end of the one concomitant vein of the DB-LCFA. Then, the thrombus was removed with a jeweler's forceps. (**C**) The removed thrombus measured 1.7 cm in length. (**D**) The ALTP flap was harvested with the whole DB-LCFA and its two concomitant veins (the yellow arrow indicates the concomitant vein affected by DVT). (**E**) The ALTP flap survived completely. DVT, deep vein thrombosis; DB-LCFA descending branch of the lateral circumflex femoral artery; ALTP, anterolateral thigh perforator.

A 70-year-old male patient presented with a massive wound in the upper half of the calf that extended to the knee area (Figure 4A). He had been injured after a traffic accident and had an open tibia fracture. Soft tissue reconstruction was performed five days post-injury. A 20 × 19 cm^2^ ALTP flap was transferred. The vascular pedicle of the ALTP flap was dissected to its origin from the LCFA and severed. However, a DVT, 1.7 cm long, was easily identified on the cutting plane of one vena comitantes (Figure 4B). This short segment of DVT was removed with a jeweler's forceps (Figure 4C), and the whole affected vein was used as a donor vein (Figure 4D). The arterial anastomosis to the ATA in an end-to-side fashion and the venous anastomosis to concomitant veins of the ATA in an end-to-end fashion were performed. The flap survived well, without any complications (Figure 4E). Anticoagulation treatment with LMWH was commenced after surgery for three months.

## Discussion

**Figure 5:**
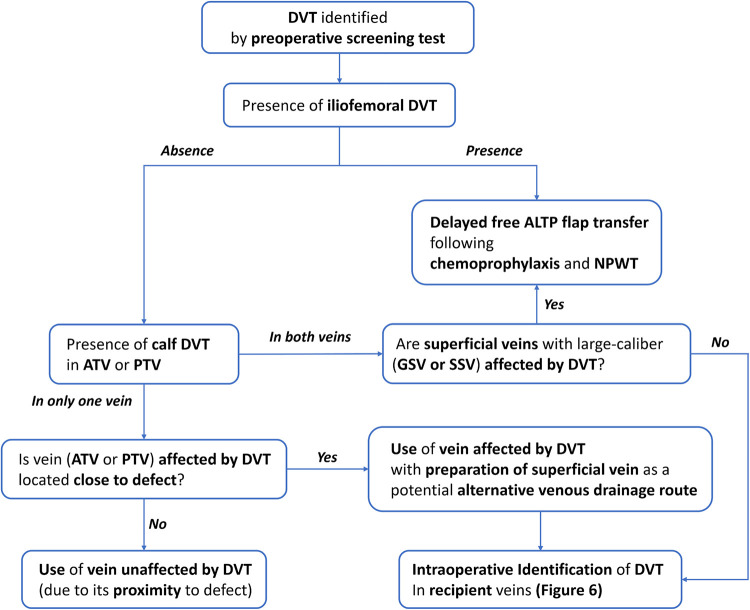
An algorithm for lower extremity reconstruction using a free ALTP flap in patients with DVT diagnosed by preoperative screening test. DVT, deep vein thrombosis; ATV, anterior tibial vein; PTV, posterior tibial vein; GSV, greater saphenous vein; SSV, small saphenous vein; NPWT, negative pressure wound therapy.

**Figure 6:**
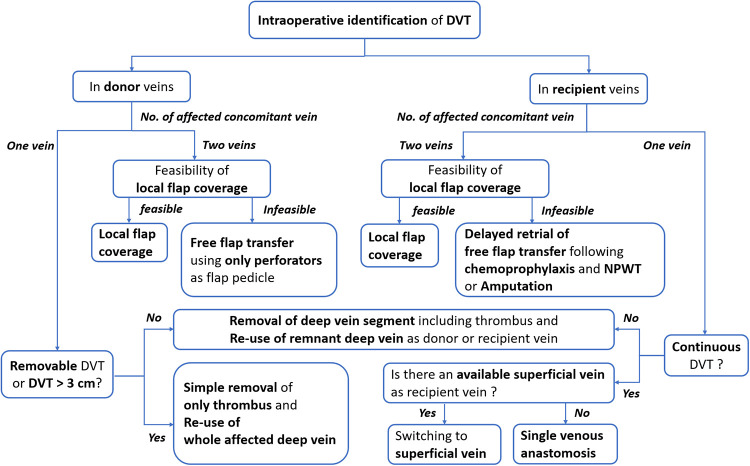
An algorithm for dealing with DVT in donor or recipient veins encountered during a free anterolateral thigh perforator flap transfer. DVT, deep vein thrombosis; NO., number; NPWT, negative pressure wound therapy.

For the free tissue transfer, deep veins were usually preferred as recipient veins due to their excellent drainage function and resistance to trauma by the protection of muscles (15). Accordingly, DVT found pre- or intraoperatively provides significant challenges to reconstructive surgeons. DVT occurring in the recipient vein located far from the anastomotic point could indirectly impair venous drainage and cause venous congestion of the transferred flap. In addition, DVT that occupied donor or recipient veins adjacent to the provisional anastomotic point could directly limit the availability of donor or recipient veins (4). Therefore, strategic surgical planning is required to minimize the negative impact of DVT on the venous anastomosis. However, there have been no guidelines to help surgeons find alternative options until now (2, 8). In this study, based on the retrospective review of our experiences, we established an algorithm to cope with DVT during free ALT flap transfer for lower leg reconstruction (Figures 5, 6).

Our algorithm has been devised based on our surgical experience only on free ALTP flap transfer. So, it may not work correctly in other free flap transfers to the lower extremities. If the recipient deep veins were shortened after resecting a thrombus-containing venous segment, or if a superficial vein should be used instead of a deep vein completely obstructed by DVT, reconstructive surgeons can flexibly deal with these changing situations with a free ALTP flap because of its long pedicle and large skin paddle. However, with other flaps, appropriate management may be impossible.

In our institution, the DVT was not screened routinely before lower extremity reconstruction using free flaps. Thus, we have two groups of patients with DVT in this study: patients diagnosed with DVT by preoperative screening CTV and patients with DVT identified intraoperatively. For these two groups, we developed two different managing algorithms based on the retrospective review of medical records. In the former group, the timing of free ALTP flap transfer could be modified depending on the locations of DVT revealed by CTV, as summarized in Figure 5. When preoperative CTV revealed that DVT occupied iliofemoral veins or both ATV and PTV simultaneously, we usually delayed surgery until the venous lumen was reestablished by recanalizing process under chemoprophylaxis. However, even though DVT obstructs both ATV and PTV on CTV, the microsurgical reconstruction may be performed successfully if superficial veins, such as great or small saphenous veins, can be used as recipient veins alternative to deep veins. Therefore, in these cases, surgery can be carried out with careful intraoperative identification of DVT in recipient veins. On the contrary, when the CTV showed DVT obstructed only one vein between ATV and PTV, the surgery was performed as planned.

In the latter group, the timing or detailed techniques of free ALTP flap transfer could be selected based on the intraoperative findings of DVT, as summarized in Figure 6. Therefore, in cases where the preoperative screening test for DVT was performed and revealed DVT, we suggest the application of the algorithm described in Figure 5 and Figure 6. in order. Contrastively, when DVT was found intraoperatively, we propose using the algorithm explained in Figure 6 only.

During free ALTP flap transfer, we always tried to use vessels close to the defect as recipient vessels, irrespective of the presence of the DVT, and achieved successful reconstructive outcomes even in cases where DVT-affected veins were used as recipient veins after appropriate manipulation using methods described in Figure 6. Thus, we do not think it is wise to adhere to using DVT-free vessels as recipient vessels even if they are far from the defect. This is because pedicle anastomosis performed far from the defect could hinder tension-free pedicle anastomosis and tension-free insetting of the skin paddle, which can lead to partial or total flap loss.

So far, there is no strong evidence in support of the relative superiority of the two venous anastomoses over one venous anastomosis for microsurgical lower extremity reconstruction. However, most experienced surgeons recommended performing two venous anastomoses if possible (16). Furthermore, the incidence of thrombosis following venous anastomosis is generally lower in deep vein anastomosis than in superficial vein anastomosis (17). Therefore, one artery and two deep veins between the flap pedicle and the recipient vessel are usually connected in free tissue transfer to the lower extremities. Two deep concomitant veins, accompanied by the artery, are popularly used for this purpose. Accordingly, the general priorities for venous anastomosis in microsurgical lower extremity reconstruction are two deep venous anastomoses, one deep and one superficial venous anastomosis, and only one deep venous anastomosis. Based on these priorities, we have transferred free ALTP flaps to the traumatic lower extremities and established an algorithmic approach (Figure 6).

Because preoperative screening tests can not accurately demonstrate DVT in each concomitant vein, DVT occurrence in these two veins should be confirmed by intraoperative investigation under magnification. Through this investigation, surgeons can identify the presence of DVT in each concomitant vein and evaluate the possibility of removing the DVT from the vein. When both concomitant veins were affected by DVT that was not removable, we discontinued free flap procedures and carried out one of three alternatives, including local flap coverage, delayed free tissue transfer, or amputation. We did not attempt to use only superficial veins as recipient veins, because we have experienced many clinical cases where deep and superficial veins were obstructed by DVT simultaneously (Figure 1D).

However, in cases with one or two concomitant veins occupied by removable DVT, we tried to re-use the vein after clearing the thrombus or resecting the venous segment containing the thrombus. Although some authors have reported that DVT could be cleared by thrombus removal using a Fogarty catheter in conjunction with massive heparin irrigation (8, 18), we have not tried catheter-based thrombectomy due to concerns about incomplete removal of organized thrombi and potential damage to the vein wall.

DVT is usually diagnosed by a screening test in asymptomatic patients. Nonetheless, routine DVT detection tests in trauma patients are not yet justified (7, 19), even in high-risk patients (20). Therefore, selective screening tests based on the risk stratification process have been recommended. However, this approach could not be applied to our patients because they were always placed in the highest risk group for DVT by risk evaluation due to severe traumatic injuries that required prolonged immobilization and a long operation. Furthermore, even if DVT was found in an early period following trauma, the use of chemical prophylaxis should be limited to avoid additional hemorrhage in patients who suffered from massive bleeding at the moment of trauma. Thus, during the early period after trauma, we just observed the occurrence of the symptoms and signs of DVT, such as unilateral lower limb swelling, pain, and redness, while applying a sequential compression device to the available leg. However, we carried out routine screening tests for DVT using CTV when the reconstructive surgery was planned seven days after the trauma to avoid postoperative DVT-related systemic complications and free flap failure.

Because there have been no guidelines for optimal timing of DVT screening tests, we established our institutional guideline for this based on previous reports and our initial experiences. Several researchers have reported that microsurgical reconstruction for traumatic lower limbs could be performed successfully within the early period following trauma. Initially, it was suggested as three days (21). However, it has been extended to about ten days (22, 23) as medical technology advances. Moreover, in most patients referred to our institution, it usually takes seven days for patients to be in stable general condition enough to avoid hemorrhagic complications following chemoprophylaxis for DVT. Besides, without the supplement of contrast media, CTV could be easily performed minutes after computed tomography angiography, which is routinely performed to evaluate the recipient arterial system prior to free ALTP flap transfer. Consequently, we decided to have a seven-day window before executing the DVT screening test.

Currently, US is the most widely used screening tool for DVT. It is safe, inexpensive, and non-invasive tool with higher sensitivity (range, 93.2%–95%) and high specificity (range, 59.8%–67.0%) for the identification of proximal DVT formed in femoral and popliteal veins. However, it has a much lower sensitivity (range, 59.8%–67.0%) for the diagnosis of distal DVT (24). In addition, performing the test could be limited by external fixators or plaster casts in patients with lower extremity trauma (25). In free ALTP flap transfer to lower extremities, not only proximal DVT but the presence of thrombus in deep veins of the lower leg, which could be employed as recipient vessels, should also be investigated adequately. In this regard, we thought the US was not an appropriate screening test for DVT detection in patients with severe lower leg trauma. Alternatively, we have used CTV as a screening test for DVT detection. CTV was relatively recently introduced to identify DVT and has produced reliable outcomes with sensitivity and specificity of 89%–100% and 94%–100%, respectively (26). CTV could identify the presence of DVT in almost all venous structures in the lower extremity and provide additional information on extravascular structures (27). However, a proper examination protocol suitable to each institutional environment should be established to obtain consistently high-quality images from various patients.

Our research is constrained by the retrospective data collection and the small number of patients with DVT that were identified during free ALTP flap transfer. However, in this study, we found that the ALTP flap could be safely transferred to traumatic lower extremities even in cases where the donor or recipient veins are obstructed by DVT. Besides, based on our experience, we developed a treatment algorithm. In the future, we plan to conduct a more extensive study on more patients with DVT prospectively to verify the usefulness of our algorithm. With further research, we will refine our algorithmic approach and produce a reliable guideline for dealing with DVT in lower extremity reconstruction using free ALTP flap transfer.

## Conclusion

DVT can be found pre- or intraoperatively during free ALTP flap transfer to traumatic lower limbs because both donor and recipient sites are located in the lower extremity, and most patients have numerous risk factors for DVT. However, since no reliable countermeasures have been reported until now, inexperienced surgeons are likely to deal with it inappropriately and fail to obtain successful outcomes. Therefore, in this study, we reviewed our experiences in this clinical setting and described our surgical solutions to deal with DVT. In addition, we developed an algorithm as a supportive tool in decision-making to find optimal alternatives to DVT-affected veins. We believe our surgical solutions and proposed algorithm can help surgeons achieve successful lower limb reconstruction using ALTP free flap even in patients with DVT.

## Data Availability

The original contributions presented in the study are included in the article/Supplementary Material, further inquiries can be directed to the corresponding author/s.
